# Factors Influencing Despair, Self-blame, and Acceptance Among Parents of Children with Autism Spectrum Disorder (ASD): A Malaysian Perspective

**DOI:** 10.1007/s10803-023-06155-8

**Published:** 2023-11-21

**Authors:** Muhammad Hanif Abd Latif, Wan Salwina Wan Ismail, Mohd Rizal Abdul Manaf, Nur Iwana Abdul Taib

**Affiliations:** 1 Department of Psychiatry, Faculty of Medicine, Universiti Kebangsaan Malaysia, Kuala Lumpur, 56000 Malaysia; 2 Hospital Canselor Tuanku Muhriz, Jalan Yaacob Latif, Bandar Tun Razak, Kuala Lumpur, 56000 Malaysia; 3 Department of Community Health, Faculty of Medicine, Universiti Kebangsaan Malaysia, Kuala Lumpur, 56000 Malaysia; 4 Department of Psychological Medicine, Faculty of Medicine and Health Sciences, Universiti Malaysia Sarawak, Kota Samarahan, 94300 Malaysia

**Keywords:** Autism, parental adjustment, Self-blame, Despair, Acceptance

## Abstract

**Purpose:**

Accepting and adapting to the child’s diagnosis of Autism Spectrum Disorder (ASD) can be challenging for parents. We aimed to assess domains of parental adjustment namely despair, self-blame, and acceptance among parents whose children were diagnosed with ASD.

**Methods:**

A cross-sectional study was conducted among 111 parents of children with autism who attended Child and Adolescent Psychiatry Unit (CAPU), in a university teaching hospital in Kuala Lumpur, Malaysia. Sociodemographic profiles of both parents and children were gathered. Parental adjustment focusing on parental self-blame, despair and acceptance were assessed using self-reported questionnaires namely Adjustment to the Diagnosis of Autism (ADA).

**Results:**

Higher level of despair was associated with parents who have medical illness (β = 0.214, p = 0.016) and children who received antipsychotic medications (β = 0.329, p < 0.001). Parents with tertiary education (β = -0.207, p = 0.023) and those with autistic child attended school (β = -0.200, p = 0.037) have lower level of despair. Parents with medical illness (β = 0.245, p = 0.008), child receiving antipsychotic medications (β = 0.251, p = 0.005), Chinese ethnicity (β = 0.185, p = 0.04), and child’s gender (β = 0.283, p = 0.003) were significantly associated with higher level of self-blame. Lower acceptance was found among Chinese parents (β = -0.264, p = 0.005) while married parents had higher acceptance levels (β = 0.215, p = 0.022).

**Conclusion:**

Parental adjustment involving domains of despair, self-blame, and acceptance were significantly associated with ethnicity of parents, educational level, parents’ marital status and medical illness, as well as the ASD children’s schooling status and type of medications used.

**Supplementary Information:**

The online version contains supplementary material available at 10.1007/s10803-023-06155-8.

Parental adjustment to ASD diagnosis is a challenging process involving important domains of despair, self-blame, and acceptance which is particularly important since it may significantly affect parental psychological well-being (Da Paz et al., 2018; Fernandez & Arcia, 2004). Despair is the feeling of hopelessness resulting from the adaptation process upon receiving the diagnosis of ASD, particularly during the early phase of the treatment. Self-blame and despair negatively influenced parental adjustment whereas acceptance served as a protective factor against mental health issues (Da Paz et al., 2018).

Parental self-blame might have originated from the social norm of poor parenting, which blamed parents for the children’s condition and behaviour, and is particularly true in the collectivist society (Francis, 2012). A qualitative study among Chinese parents of children with ASD revealed that parents were frequently criticized for the failure to control the behavioural problem displayed by the children in public, hence labelled as incompetent parents (Ng, 2022). Self-stigma might cause parents to place the responsibility on themselves, leading to self-blame (Colic et al., 2019). Parental self-blame could also be attributed to the parents’ lack of knowledge, affecting the parents’ understanding of the behavioural issues among children with ASD (Crane et al., 2016). It had been demonstrated that parental self-blame was decreased after seeking professional guidance and support (Legg & Tickle, 2019). Interestingly, a study had shown that parental despair among parents of ASD children was higher compared to parents of children with other neurodevelopmental disorders due to a lower level of acceptance of the diagnosis of ASD. (Zembat & Yildiz, 2010).

Lack of knowledge of ASD in society, particularly in Malaysia contributed significantly to the increasing stigmas (Yaacob et al., 2022). Furthermore, cultural influence holds parents to be responsible for children’s diagnosis and consequently affects parental adjustment (Mak & Kwok, 2010; Sarrett, 2015). According to a Malaysian study, parents were able to accept the diagnosis of ASD after shifting the perspective from a crisis or stress, to one of gratitude and positive appreciation (Chu et al., 2020). Parents’ religious beliefs also helped them to accept the diagnosis in a more positive light (Yaacob et al., 2022). The child’s age, the severity of the diagnosis, the parents’ gender, parents’ level of education, and the duration of time since diagnosis, were significant factors affecting parental acceptance level (Poslawsky et al., 2014; Yirmiya et al., 2015). Another study linked parental despair to the incapability of parents in handling the behavioural problem of the ASD children (Kalaivnai & Kalimo, 2018). As they were informed of the diagnosis, they adopted a mindset of learned helplessness and believed that nothing could be changed (Myers et al., 2009). However, the feeling of hopelessness can be improved with the adaptation of good coping skills by the parents such as problem-focused parenting techniques which would increase parenting effectiveness, reduce parental despair, and make parents to be more optimistic about the future (Pozo et al., 2014).

Parental adjustment upon learning the diagnosis of ASD in children is important as it has a big impact on the mental health. While accepting the diagnosis of ASD could be difficult, some parents were more resilient in accepting the diagnosis (Bonis, 2016; Zhao & Fu, 2020). Importantly, carers who experienced higher levels of self-blame and despair saw a decline in mental health and degree of life satisfaction (Da Paz et al., 2018). Parents’ self-blame and self-stigma were significantly correlated, caused emotional distress and depressive symptoms among parents (Eaton et al., 2016; Manan et al., 2018), and predicted parental happiness in parenting the children (Findler et al., 2016). Parental stress can also be explained through the parent-child transactional relationship model where both parents’ and children’s emotions or behaviors influences each other in a reciprocate manner (Kerr et al., 2012). A study on transactional relations by Neece et al. (2012) found that parenting stress served as both a trigger and a result of child behavior difficulties while child behavior problems simultaneously served as a trigger and a result of parenting stress (Neece et al., 2012).

Unfortunately, there is a dearth of research on parental adjustment among parents of children with ASD globally. This is concerning given the importance of parental adjustment and its significant impact on mental health. Therefore, this study aimed to assess domains of parental adjustment namely self-blame, despair, and acceptance among parents whose children were diagnosed with ASD. It is hoped that this study would add new insight and facilitate clinicians in supporting parental adjustment during and after the delivery of ASD diagnosis.

## Materials and Methods

### Study Design and Respondents

This is a cross-sectional study conducted among parents of patients with ASD, attending Child and Adolescent Psychiatry Unit (CAPU), in a university teaching hospital in Kuala Lumpur, Malaysia. Parents were approached during the clinic sessions and conveniently recruited. Those who missed the clinic appointments were approached via phone calls and text messages. The inclusion criteria were: (1) parents who had a child or children diagnosed with ASD by the child and adolescent psychiatrist using the DSM5 criteria and confirmed using the Autism Spectrum Disorder Module for the Mini International Neuropsychiatric Interview (MINI) 7.0.2, (2) parents who lived with the child or children (3) parents who were able to read, write and understand Malay or English and (4) consented to participate.

### Data Collection and Measures

Both manual and online questionnaires were used in this study. Parents who attended clinic sessions were asked to answer the questionnaires manually, whereas parents who were absent completed the questionnaires online. Sociodemographic profiles of both parents and children, as well as clinical characteristics, were gathered. Parental adjustments were assessed using Adjustment to Diagnosis of Autism (ADA), a 30-item self-reported questionnaire focusing on parental self-blame, parental despair, and parental acceptance (Da Paz et al., 2018). Parents were asked to rate the degree of agreement with statements regarding the child’s autism diagnosis using a 4-point Likert scale ranging from 1 (Don’t agree at all) to 4 (very strongly agree). The higher score indicates the higher level of despair, self-blame, and acceptance among parents following children’s diagnosis of ASD. The inference can also be made by comparing the mean score to the maximum score. The questionnaire was translated into the Malay language with permission from the original author. Although it was not validated, it showed good internal consistency with Cronbach alpha of 0.7 to 0.8 for both English and the Malay language. Both English and Malay versions of ADA were used in the study according to the preferences of respondents.

A total of 111 parents participated in the study, which was slightly higher than the calculated sample size of 106, using the formula of ‘comparing two mean formula’ by Whitley & Ball (2002) for sample size which gave 90% power of the study (Norizan & Shamsuddin, 2010). The sociodemographic and clinical characteristics of respondents and the children were summarized in Table 1(a) and 2(b). Majority of the participating parents were Malays (73.9%), females (67.6%), married (93.7%), had tertiary education (73%), middle-tier household or M40 (RM4850 – RM10970) (39.6%), employed (69.4%), and had no psychiatric illness (95.5) or medical illness (73%). Meanwhile, 86% of the children did not have sibling with ASD. Most of them had attended EIP (67.6%) and/or school (89.2%). There was only 9% of the children were reported to have comorbid psychiatric illness and majority of them were not on medications (78.4%).

**Table 1:** **(a)** Sociodemographic factors of parents of children with ASD

Variables	Number of participants (n = 111)	Percentage (%)
Age (parents) Gender (parents):	44.0^#^	
Male	36	32.4
Female	75	67.6
Ethnicity:		
Malay	82	73.9
Chinese	21	18.9
Indian	3	2.7
Others	5	4.5
Employment status:		
Employed	77	69.4
Unemployed / Housewife	34	30.6
Monthly household income (DOSM 2020):		
B40 (< RM4850)	39	35.1
M40 (RM4850 – RM10970) T20 (> RM10970)	44 28	39.6 25.2
Marital status:		
Married	104	93.7
Divorced	7	6.3
Education status:		
Primary education	4	3.6
Secondary education	26	23.4
College / University	81	73.0
Parents with psychiatry illness:		
Yes	5	4.5
No	106	95.5
Parents with medical illness:		
Yes	30	27.0
No	81	73.0

# = mean in years

**Table 1:** **(b)** Sociodemographic factors of children with ASD

Variables	Number of participants (n = 111)	Percentage (%)
Age (Child) Gender (Child):	13.18^#^	
Male	88	79.3
Female	23	20.7
Other siblings with ASD:		
Yes	15	13.5
No	96	86.5
Attended EIP:		
Yes	75	67.6
No	36	32.4
Attended school:		
Yes	99	89.2
No	12	10.8
Underlying medical illness:		
Yes	14	12.6
No	97	87.4
Underlying other psychiatric illness:		
Yes	10	9.0
No	101	91.0
Current medication:		
No medication	87	78.4
Antipsychotics	12	10.8
Stimulants (Eg: Methylphenidate)	6	5.4
Multiple medications	6	5.4

# = mean in years, EIP = Early Intervention Program

All data were analyzed using the IBM SPSS Statistics (Version 27). Data were normally distributed based on the normal Q-Q Plots, skewness, and kurtosis values. Parental self-blame, despair, and acceptance were reported as mean scores. The association between ADA domains (i.e. parental self-blame, despair, and acceptance), sociodemographic and clinical characteristics were analyzed using independent sample t-test and one-way analysis of variance (ANOVA). Subsequently, the significant variables were further analyzed with multiple linear regression to control for any possible confounder statistically. The statistical significance was p < 0.05.

## Results

The supplementary materials contain details of the sociodemographic and clinical characteristics that exhibited statistical significance following analysis through both independent samples t-test and ANOVA. Meanwhile, associations between parental despair and other variables were presented in Table 2. Parental despair showed statistically significant association between parents’ educational levels (β = -0.207, p = 0.023) parents’ medical illnesses (β = 0.214, p = 0.016), schooling status of ASD children (β = -0.200, p = 0.037), and being on antipsychotics (β = 0.329, p = < 0.001). Highly educated parents and ASD children who attended school were protective against parental despair. In contrast, parents who had medical illnesses and ASD children on antipsychotics increased risk for parental despair. The multiple linear regression model showed a low R^2^ of 0.278.

**Table 2:** Associations between parental adjustment (despair) and other variables among parents of children with diagnosis of ASD

Variables	Mean Score		Standard Deviation		Standardized Coefficient Beta	95% Confidence Interval	P-Value
Parents’ education level:							
Primary school		32.75		5.909	Reference		
Secondary school		26.12		9.262	-0.056	-9.935 to 5.705	0.593
College / University		23.68		6.004	-0.207	-6.133 to -0.459	0.023*
Parents with medical illness:							
Yes		27.73		8.432	0.214	0.658 to 6.146	0.016*
No		23.41		6.180	Reference		
Child Attending School:							
Yes		23.93		7.076	-0.200	-8.813 to -0.275	0.037*
No		29.92		4.641	Reference		
Child current medications:							
No medication		23.23		6.283	Reference		
Antipsychotics		31.67		7.050	0.329	3.525 to 11.413	< 0.001*
Stimulants		28.67		10.820	0.126	-1.352 to 9.208	0.143
Multiple medications		25.83		5.076	0.089	-2.612 to 8.154	0.310

The model was significant at the 0.05 level, F = 5.666, p < 0.001, R^2^ = 0.278

The association between parental self-blame and other variables were presented in Table 3. Based on the multiple linear regression model (R^2^ = 0.231), Chinese parents (β = 0.185, p = 0.040), parents with medical illness (β = 0.245, p = 0.008), male gender of ASD child (β = 0.283, p = 0.003) and ASD child on antipsychotics (β = 0.251, p = 0.005) were significant risk factors for parental self-blame.

**Table 3:** Associations between parental adjustment (self-blame) and other variables among parents of children with diagnosis of ASD

Variables	Mean Score	Standard Deviation	Standardized Coefficient Beta	95% Confidence Interval	P-Value
Race:					
Malay	24.70	5.300	Reference		
Chinese	27.76	5.098	0.185	0.123 to 4.903	0.040*
Indian	23.67	5.132	-0.082	-8.603 to 3.252	0.373
Gender of child:					
Male	26.00	4.994	0.283	1.330 to 6.093	0.003*
Female	23.39	6.207	Reference		
Parents with medical illness:					
Yes	27.37	6.003	0.245	0.744 to 5.086	0.008*
No	24.75	4.933	Reference		
Child current medications:					
No medication	24.71	5.119	Reference		
Antipsychotics	29.92	4.441	0.251	1.313 to 7.302	0.005*
Stimulants	27.00	8.099	0.049	-3.085 to 5.382	0.592
Multiple medications	25.83	2.858	0.073	-2.465 to 5.879	0.419

The model was significant at the 0.05 level, F = 4.414, p < 0.001, R^2^ = 0.231

Table 4 presented the association between parental acceptance and other variables. Parental acceptance showed significant association with parents’ marital status and ethnicity. Married parents (β = 0.215, p = 0.022) and Chinese parents (β = 0.264, p = 0.005) were significantly associated with parental acceptance. The multiple linear regression model had low R^2^ of 0.119.

**Table 4:** Associations between parental acceptance and other variables among parents of children with diagnosis of ASD

Variables	Mean Score	Standard Deviation	Standardized Coefficient Beta	95% Confidence Interval	P-Value
Race:					
Malay	21.29	2.521	Reference		
Chinese	19.57	3.026	-0.264	-3.045 to -0.572	0.005*
Indian	19.67	3.215	-0.063	-4.073 to 2.003	0.501
Marital status:					
Married	21.13	2.679	0.215	0.351 to 4.393	0.022*
Divorced	18.71	1.890	Reference		

The model was significant at the 0.05 level, F = 4.801, p = 0.004, R^2^ = 0.119

## Discussion

This study found significant associations between parental adjustment and various sociodemographic and clinical factors. Concerning parental despair, highly educated parents and ASD children who attended school were protective against parental despair. These findings were in keeping with previous study that explained the possibility of highly educated parents having a better understanding of the diagnosis which eventually helped them to be more hopeful about the child’s condition (Vetrayan et al., 2013). On the other hand, parents whose children were attending school reported lower level of parental despair. In this context, the school could be viewed as a strong support system for parents to share the caregiving burden, which resulted in lower level of parental despair (Foronda, 2000; Seeridaram & Rashid, 2023). School also provided structure and routine that ASD children needed, indirectly reducing behavioural problems among the children and the level of despair among parents.

In support of previous findings, parents who had underlying medical illness was at risk of developing parental despair (Reed & Osbourne, 2019). A previous study found parents who had physical illness, showed poor adaptation to the diagnosis of ASD (Reed & Osbourne, 2019), potentially leading to high level of parental despair. Having ASD children who were prescribed antipsychotic medications, was another risk factor for parental despair. In our study, the number of children with ASD who were prescribed antipsychotics is 10.8% which is lower than expected prevalence of 17% (Jobski et al., 2017). It is postulated that this group of children on antipsychotics might have severe behavioural issues, given that antipsychotics were indicated pharmacological treatment for challenging behaviour in children with ASD (Park et al., 2016). Therefore, it is likely that these children might have severe ASD features, which were directly associated with parental despair (Lai et al., 2015).

With regards to the other domains of parental adjustment, Chinese parents reported significant high self-blame and low acceptance of the children’s diagnosis of ASD. It has been shown in the previous study that ethnicity is a significant moderator in the relationship between family resilience and parenting stress among caregivers of ASD children (Kim et al., 2020). Given the strong collectivist element of chinese culture, any misbehaviour of the children is normally blamed on the parents. As such, it might explain the higher tendency of Chinese parents to self-blame, and consequently lower acceptance level (Zhao & Fu, 2020). Furthermore, there is a strong cultural belief among Chinese families that the previous wrongdoing were the cause of ASD in the children (Ilias et al., 2017), hence the tendency to self-blame. In the same cultural context, problematic behaviours among children commonly receive negative public perception and are frequently blamed on poor parenting, leading to increase in self-blame (Kwok et al., 2014; Whitehead et al., 2015; Papadopoulos et al., 2019). Having medical illness would further compromise the physical and psychological health, consequently leading to higher self-blame (Schulz & Sherwood, 2008).

Parental acceptance was significantly low among chinese parents, which was understandable given the significantly higher level of despair and self-blame among them. The role of culture in perceiving and accepting the diagnosis of ASD has been explored by several studies in the past (Lee et al., 2017; Ha et al., 2014). Cultural factor influenced parental ability to attribute the diagnosis of ASD to karma, parental sins, or curses, rather than looking at it from the medical perspective, hence the difficulty to accept the ASD diagnosis (Ilias et al., 2017).

In keeping with previous findings, this study found married parents had higher acceptance of ASD diagnosis compared to single or divorced parents. Spouse support is therefore important to influence and improve parental acceptance of the ASD diagnosis (Santoso et al., 2015; Chong et al., 2016; Mohamad Aun et al., 2022). This could also be due to better communication between spouses regarding the child’s condition leading to increased spousal support and sense of partnership in caregiving (Marciano et al., 2015).

The main strength of our study is its novelty in exploring parental adjustment and acceptance, and its association with sociodemographic and clinical characteristics, providing new insight to our understanding of these aspects of ASD. To the best of our knowledge, this is among the first study to assess parental level of despair, self-blame, and acceptance in relation to diagnosis of ASD. Such understanding will support clinicians to facilitate parents in improving their ability to positively adapt to diagnosis of ASD in their children.

However, the finding of this study need cautious interpretation given its limitations. Firstly, the findings may not be generalized to normal population due to the non-probability sampling of the urban area. This population might have better understanding of ASD due to easier access to information regarding the subject matter. On the other hand, parents in rural or sub-urban settings might have lack of support from other agencies to reduce their burden of care and limited resources for educational support. Secondly, we did not analyze result for mothers and fathers separately due to the very limited number of mothers and fathers of the same child with ASD that participated in the study. This is important because of the possible significant differences in between each parent’s levels of adjustment, acceptance, and parental stress. Thirdly, all the instruments were self-reported questionnaires which may introduce recall bias in the study. Finally, the cross-sectional nature of the study does not allow causal association to be determined. Moving forward, it is important to explore the association between the level of parental adjustment and acceptance of children with ASD, and the level of parental distress. A longitudinal study could also help to ascertain the causal relationship between the variables and give a better view about this topic.

In conclusion, parental adjustment involving domains of despair, self-blame, and acceptance were significantly associated with ethnicity of parents, educational level, marital status and medical illness, as well as the ASD children’s schooling status and being on antipsychotics. Perceived from the local cultural context, these new understanding provide valuable insight to facilitate clinicians in supporting parents through the adjustment process upon receiving the diagnosis of children with ASD.

## Electronic supplementary material

Below is the link to the electronic supplementary material.

Supplementary Material 1

## Declarations

This study was approved by the Medical Research Committee of the hospital (UKM FPR.SPI 800-2/27/FF-2021-399) and it abides by the regulations of the 1964 Declaration of Helsinki and its subsequent amendments.
