# Supplementary material for: Factors Influencing Despair, Self-blame, and Acceptance Among Parents of Children with Autism Spectrum Disorder (ASD): A Malaysian Perspective
Source: J Autism Dev Disord. 2023 Nov 21;55(6):2067–74. doi: 10.1007/s10803-023-06155-8 (PMC12069508; doi:10.1007/s10803-023-06155-8)
Supplement: Supplementary file 1 — Supplementary Material 1 [file 10803_2023_6155_MOESM1_ESM.docx]

**SUPPLEMENT MATERIAL**

Table 1(S). Association between parental despair and ASD child schooling status

| Variables | Mean Score | | | SD | t value | P-Value |
| --- | --- | --- | --- | --- | --- | --- |
| Child Attending School: | | |  | |  |  |
| Yes | | 23.93 | 7.08 | | 2.851 | 0.005* |
| No | | 29.92 | 4.64 | |  |  |

Table 2(S). Association between parental despair and parents’ medical illness

| Variables | Mean Score | | | SD | t value | P-Value |
| --- | --- | --- | --- | --- | --- | --- |
| Parents’ Medical Illness: | | |  | |  |  |
| Yes | | 27.73 | 8.43 | | 2.954 | 0.004* |
| No | | 23.41 | 6.18 | |  |  |

Table 3(S). Association between parental despair and child’s current medication

| Variables | Mean Score | | | SD |
| --- | --- | --- | --- | --- |
| Child current medications: | | |  | |
| No medication | | 23.23 | 6.283 | |
| Antipsychotics | | 31.67 | 7.050 | |
| Stimulants | | 28.67 | 10.820 | |
| Multiple medications | | 25.83 | 5.076 | |

The ANOVA was significant at the 0.05 level, F = 6.671, p < 0.001

Table 4(S). Association between parental despair and parents’ educational level

| Variables | Mean Score | | | SD |
| --- | --- | --- | --- | --- |
| Child current medications: | | |  | |
| Primary education | | 32.75 | 5.909 | |
| Secondary education | | 26.12 | 9.262 | |
| College/University | | 23.68 | 6.004 | |

The ANOVA was significant at the 0.05 level, F = 4.145, p = 0.018

An independent-samples t-test was conducted to compare the parental despair scores for children attending school and not attending school (table 1(S)). There was a significant difference in score for parental despair scores for children attending school (mean = 23.93, s.d.= 7.08) and parental despair scores for children not attending school (mean = 29.92, s.d.= 4.64), with t-value of 2.851 and p value of 0.005. An independent-samples t-test was conducted to compare the parental despair scores for parents who have medical illness and do not have medical illness as presented in Table 2(S). There was a significant difference in score for parental despair scores for parents who have medical illness (mean = 27.73, s.d.= 8.43) and parental despair scores for parents who do not have medical illness (mean = 23.41, s.d.= 6.18), with t-value of 2.95 and p value of 0.004. An ANOVA was conducted to explore the impact of child’s current medication on the score of parental despair. The children were divided into four groups according to medications status (no medication; on antipsychotics; on stimulants and on multiple medications). The means and standard deviations are presented in Table 3(S). There was a statistically significant difference in the parental despair for the four groups (F = 6.671, p < 0.001). Post-hoc comparisons using the Tukey HSD test indicated that the mean score of parental despair for the children who were on antipsychotic (mean = 31.67, s.d = 7.050) was significantly different from the children who were not on medication (mean = 23.23, s.d = 6.283). An ANOVA was conducted to explore the impact of parents’ educational status on the score of parental despair. The parents were divided into three groups according to educational status (primary education; secondary education; and college/university). The means and standard deviations are presented in Table 4(S). There was a statistically significant difference in the parental despair for the three groups (F = 4.145, p = 0.018). Post-hoc comparisons using the Tukey HSD test indicated that the mean score of parental despair for parents with only primary education (mean = 32.75, s.d = 5.909) was significantly different from parents who attended college/university (mean = 23.68, s.d = 6.004).

Table 5(S). Association between parental self-blame and race

| Variables | Mean Score | | | SD |
| --- | --- | --- | --- | --- |
| Race: | | |  | |
| Malay | | 24.70 | 5.300 | |
| Chinese | | 27.76 | 5.098 | |
| Indian | | 23.67 | 5.132 | |

The ANOVA was significant at the 0.05 level, F = 3.037, p = 0.032

Table 6(S). Association between parental self-blame and gender of child

| Variables | Mean Score | | | SD | t value | P-Value |
| --- | --- | --- | --- | --- | --- | --- |
| Gender of child: | | |  | |  |  |
| Male | | 26.00 | 4.99 | | 2.117 | 0.037* |
| Female | | 23.39 | 6.21 | |  |  |

Table 7(S). Association between parental self-blame and parents with medical illness

| Variables | Mean Score | | | SD | t value | P-Value |
| --- | --- | --- | --- | --- | --- | --- |
| Parents with medical illness: | | |  | |  |  |
| Yes | | 27.37 | 6.00 | | 2.334 | 0.021* |
| No | | 24.75 | 4.93 | |  |  |

Table 7(S). Association between parental self-blame and child’s current medication

| Variables | Mean Score | | | SD |
| --- | --- | --- | --- | --- |
| Child current medications: | | |  | |
| No medication | | 24.71 | 5.119 | |
| Antipsychotics | | 29.92 | 4.441 | |
| Stimulants | | 27.00 | 8.099 | |
| Multiple medications | | 25.83 | 2.858 | |

The ANOVA was significant at the 0.05 level, F = 3.793, p = 0.012

An ANOVA was conducted to explore association between race on the score of parental self-blame. The participants were divided into three groups according to their race (Malay; Chinese; Indian). The means and standard deviations are presented in Table 5(S). There was a statistically significant difference in the parental self-blame for the three groups (F = 3.037, p = 0.032). However, Post-hoc comparisons using the Tukey HSD test did not indicated any significant different between the three ethnic groups. An independent-samples t-test was conducted to compare the parental self-blame scores between gender of the child as presented in Table 6(S). There was a significant difference in score for parental self-blame scores for male ASD child (mean = 26.00, s.d.= 4.99) and female ASD child (mean = 23.39, s.d.= 6.21), with t-value of 2.117 and p value of 0.037. An independent-samples t-test was conducted to compare the parental self-blame scores for parents who have medical illness and do not have medical illness as presented in Table 7(S). There was a significant difference in score for parental self-blame scores for parents who have medical illness (mean = 27.37, s.d.= 6.00) and parental despair scores for parents who do not have medical illness (mean = 24.75, s.d.= 4.93), with t-value of 2.334 and p value of 0.021. An ANOVA was conducted to explore the impact of child’s current medication on the score of parental self-blame. The children were divided into four groups according to medications status (no medication; on antipsychotics; on stimulants and on multiple medications). The means and standard deviations are presented in Table 8(S). There was a statistically significant difference in the parental self-blame for the four groups (F = 3.793, p = 0.012). Post-hoc comparisons using the Tukey HSD test indicated that the mean score of parental self-blame for the children who were on antipsychotic (mean = 29.92, s.d = 4.441) was significantly different from the children who were not on medication (mean = 24.71, s.d = 5.119).

Table 8(S). Association between parental acceptance and race

| Variables | Mean Score | | | SD |
| --- | --- | --- | --- | --- |
| Race: | | |  | |
| Malay | | 21.29 | 2.521 | |
| Chinese | | 19.57 | 3.026 | |
| Indian | | 19.67 | 3.215 | |

The ANOVA was significant at the 0.05 level, F = 3.155, p = 0.028

Table 9(S). Association between parental acceptance and parents’ marital status

| Variables | Mean Score | | | SD | t value | P-Value |
| --- | --- | --- | --- | --- | --- | --- |
| Parents’ marital status: | | |  | |  |  |
| Yes | | 21.13 | 2.68 | | 2.337 | 0.021* |
| Divorced | | 18.17 | 1.89 | |  |  |

An ANOVA was conducted to explore association between race on the score of parental acceptance. The participants were divided into three groups according to their race (Malay; Chinese; Indian). The means and standard deviations are presented in Table 8(S). There was a statistically significant difference in parental acceptance for the three groups (F = 3.155, p = 0.028). Post-hoc comparisons using the Tukey HSD test indicated that the mean score of parental acceptance for Malay (mean = 21.29, s.d = 2.521) was significantly different from Chinese ethnicity (mean = 19.57, s.d = 3.026). An independent-samples t-test was conducted to compare the parental acceptance scores for married and divorced as presented in Table 7(S). There was a significant difference in score for parental acceptance scores for married parents (mean = 21.13, s.d.= 2.68) and parental acceptance scores for divorced parents (mean = 18.17, s.d.= 1.89), with t-value of 2.337 and p value of 0.021.
